# Hepatitis B Infection in Microbiology Laboratory Workers: Prevalence, Vaccination, and Immunity Status

**DOI:** 10.1155/2012/520362

**Published:** 2012-12-04

**Authors:** Arun Kumar Jha, Sanjim Chadha, Preena Bhalla, Sanjeev Saini

**Affiliations:** ^1^Department of Microbiology, Maulana Azad Medical College, University of Delhi, Bahadur Shah Zafar Marg, New Delhi 110002, India; ^2^Central Surveillance Unit, Integrated Disease Surveillance Project (IDSP), National Center for Disease Control (NCDC), New Delhi 110054, India

## Abstract

The risk of contracting HBV by health care workers (HCW) is four-times greater than that of general adult population. Studies have demonstrated that vaccine-induced protection persists at least 11 years. High risk groups such as HCWs should be monitored and receive a booster vaccination if their anti-HBsAb levels decrease below 10 mIU/mL. In view of the above this study was undertaken to assess the HBV vaccination of the HCWs and their immunological response. Seventy-two HCWs of the Department of Microbiology, Maulana Azad Medical College, New Delhi, India, were recruited and blood sample was drawn for serological tests (HBSAg, anti-HCV, anti-HBsAb, anti-HBeAb, and anti-HBcAb). Anti-HBs titers of >10 mIU/mL were considered protective. Thirty-four (47.3%) of the participants were completely vaccinated with three doses. 25 (73.5%) of the participants with complete vaccination had protective anti-HBsAb levels as against 8 (53.3%) of those with incomplete vaccination and 9 (39.1%) of those who were not vaccinated at all. One of our participants was acutely infected while 29 participants were susceptible to infection at the time of the study. All HCWs should receive three doses of the vaccine and be monitored for their immune status after every five years. Boosters should be administered to those who become susceptible.

## 1. Introduction

Hepatitis B is a serious infectious disease of the liver which affects millions of people worldwide. More than 2 billion people living today have been infected with Hepatitis B virus (HBV) at some time in their lives and about 350 million people are carriers of the virus [1]. India has intermediate endemicity of Hepatitis B, with Hepatitis B surface antigen (HBsAg) prevalence between 2% and 7%. The number of HBsAg carriers in India has been estimated to be over 50 millions [2, 3].

Throughout the world, millions of healthcare professionals work in health institutions and it is estimated that 600,000 to 800,000 cut and puncture injuries occur among them per year, of which approximately 50% are not registered [4]. The risk of contracting HBV by health care workers (HCWs) is four-times greater than that of general adult population [5]. The highest rates are seen among dentists, physicians, laboratory workers, dialysis workers, cleaning service employees, and nurses [6].

Blood contains the highest HBV titers of all body fluids and is the most important vehicle of transmission in the health care setting. Avoiding occupational blood exposure is the primary preventive means for the transmission of HBV. Immunization and after exposure management are integral components of a complete infection control program for this group [7].

Protection (defined as antihepatitis B virus surface antigen antibodies (anti-HBsAb) level ≥10 mIU/mL) following first, second, and third doses of the recombinant vaccine has been reported to be 20–30%, 75–80%, and 90–95%, respectively [8, 9]. Studies have demonstrated that vaccine-induced protection persists at least 11 years and booster vaccination for immunocompetent children and adults is not recommended for long-term protection [10, 11]. Immunocompromised patients and high risk groups such as health care workers, however, should be monitored and receive a booster vaccination if their anti-HBsAb levels decrease below 10 mIU/mL [12, 13].

In our study we determined the HBV serological status of microbiology laboratory workers in a tertiary care hospital in New Delhi, India. We also analyzed the HBV vaccination of the laboratory workers and their long-term immunological response to vaccination in view of administering a booster dose to those with anti-HBsAb levels <10 mIU/mL. 

## 2. Materials and Methods

This study was conducted in the Department of Microbiology, Maulana Azad Medical College, New Delhi, India. Seventy-two participants were enrolled for the study in the age group 20–70 years. Participants consisted of Professors, Resident Doctors, Ph.D. scholars, laboratory technicians and laboratory attendants working in the Department of Microbiology. After obtaining an informed written consent from each participant, all laboratory workers were asked to complete a questionnaire consisting of their age, gender, Hepatitis B vaccination status, their job description, and educational level.

Blood sample was drawn from each participant under strict aseptic precautions in a plain vaccutainer. Blood was allowed to clot and serum was separated and stored at −20°C until further testing. Serological tests (HBSAg, antihepatitis C virus antibodies (anti-HCV), anti-HBsAb, anti Hepatitis B virus e antigen antibody (anti-HBeAb), and anti Hepatitis B virus core antigen antibody (anti-HBcAb)) were performed using commercially available ELISA kits according to the manufacturer's instructions. Anti-HBs titers of >10 mIU/mL were considered protective.

## 3. Results

A total of 72 participants were recruited including 40 males (55.6%) and 32 females (44.4%) with a male female ratio of 1.25 : 1. The median age of study subjects was 31.5 years (range 20–70 years) with 20–40 years being the most common age group. Fifteen (20.8%) of our participants were laboratory attendants, 21 (29.2%) were laboratory technicians, 10 (13.8%) were Ph.D. scholars, 18 (25%) were resident doctors and 4 (5.6%) were Professors in Department of Microbiology. Thirty-four (47.3%) of the participants were completely vaccinated for hepatitis B with three doses of the vaccine while 15 (20.8%) of them had not completed the vaccination schedule, and 23 (31.9%) were not vaccinated at all (Table 1). 

Twenty-five (73.5%) of the participants with complete vaccination had protective anti-HBsAb levels as against 8 (53.3%) of those with incomplete vaccination and 9 (39.1%) of those who were not vaccinated had protective anti-HBsAb levels (Table 2; *P* value = 0.032).

Out of the 34 subjects who were completely vaccinated, 2 had received vaccination <5 years ago, 17 were vaccinated 5–10 years ago and 15 were vaccinated >10 years before the study. High titers of anti-HBsAb (>100 mIU/mL) were seen in 100% of the cases in the first group (<5 years since vaccination) and 41.2% and 33.4% in the other two groups, respectively (Table 3; *P* value = 0.071).

Table 4 shows the status of vaccination in relation to the professional designation in the department. 59.4% of our staff with a medical degree was completely vaccinated as against 37.5% of other staff in the department.

Table 5 shows the hepatitis B serologic tests of all study subjects (*n* = 72). Twenty-nine (40.3%) participants were susceptible to infection at the time of the study irrespective of their vaccination status. Seven (9.8%) were immune due to natural infection (recent and remote) while 35 (48.6%) were immune due to hepatitis B vaccination. Only one of our participants was acutely infected at the time of the study. Three (4.2%) had isolated positive anti-HBcAb with negative HBs Ag, anti-HBsAb, and anti-HBeAb. None of the participants demonstrated positive anti-HCV at the time of the study.

## 4. Discussion

Vaccination is an important measure in preventing HBV infection in health care workers. In our study only 47.3% had received three doses of hepatitis B vaccine, while 20.8% had incomplete vaccination and 31.9% were not vaccinated at all. These observations indicate that hepatitis B vaccination coverage is still inadequate in our country even among high risk groups such as laboratory workers. The main reason behind this could be due to an absence of vaccination policies established by the hospital management and also a lack of awareness or inclination on the part of Laboratory workers. Surprisingly even in some developed countries like Sweden, only 40% health care workers reported that they were fully vaccinated and 21% had not been vaccinated at all [14].

An interesting observation in our study was that 59.4% of the doctors had received complete vaccination while 37.5% of other staff (laboratory attendants, assistants, and technicians) was completely vaccinated (Table 4; *P* value = 0.136). This contrast in vaccination coverage can be explained by the difference in education and awareness among the two groups. 28.1% of the doctors were not vaccinated at all and this is due to the fact that this group also includes Ph.D. scholars who have not yet worked in a medical hospital before joining this degree course and therefore do not feel the need to get themselves vaccinated. In a study done in AIIMS, New Delhi, 96% doctors were vaccinated [15]. 

The prevalence of acute HBV infection (HBsAg positive) in our Laboratory workers was found to be 1.4% (1/72). This person was a 58-year-old laboratory technician. However, since he was not directly involved in collection of blood or serological testing of blood samples, his infection may not have been acquired by him due to his occupation. The percentage positivity of HBsAg in another study conducted in GB Pant Hospital, New Delhi was found to be 1% [16].

Twenty-five (73.5%) of our study participants with complete vaccination had protective (>10 mIU/mL) anti-HBsAb levels as against 8 (53.3%) of those with incomplete vaccination and 9 (39.1%) of those who were not vaccinated had protective anti-HBsAb levels. These findings show that 26.5% of the laboratory workers with complete vaccination had not achieved protective antibody levels and were still susceptible to acquire infection. These workers were asked to take a booster dose of hepatitis B vaccine. However 39.1% of those who were not vaccinated at all attained protective antibody levels probably due to the acquisition of natural infection sometime in the past which conferred natural immunity to these individuals. Another important observation that our study highlights is that 40.3% of our laboratory workers were not immune and were still at risk of hepatitis B infection. This again points to the lapse of the higher authorities for not creating a vaccination strategy for laboratory workers and also not increasing awareness among health care workers for getting them vaccinated. Our findings are similar to a study conducted on nursing students in a tertiary care hospital in Chandigarh in which of the ones who had received a complete course of HBV vaccination 82.2% showed protective levels. The protective anti-HBs antibody levels in students who were unvaccinated or where vaccination status was not known were 36% and 29% respectively [17].

Three (4.2%) of our study participants showed isolated positive anti-HBc serological reaction. This could be due to a false positive anti-HBc reaction or subjects being in the window period of infection. False positive anti-HBc results may occur when nonspecific IgM binds to the HBcAg peptides used as a probe in the assay. As with any screening assay, the anti-HBc assay has greater specificity in high risk populations. Thus, if a patient with risk factors for hepatitis B infection demonstrates isolated anti-HBc in serum, a false positive result would be less likely than in a patient without riskfactors but the possibility still exists [18, 19]. These cases were referred for HBV DNA testing and IgM anti-HBcAb testing to determine whether they were in the window phase, or have occult HBV infection. A study conducted in a Brazilian university hospital in 2005 showed that 9.4% of HCWs had positive anti-HBc [20].

In our study, protective anti-HBsAb titers were seen in 100% of the cases who were completely vaccinated <5 years before the study and in 58.8% and 86.6% of those who had received a complete course of vaccination 5–10 years and >10 years ago, respectively. Another study from Iran has shown that 89% of subjects with complete vaccination less than five years before achieved protective anti-HBs titers and 13.9% cases who had been vaccinated within 5–10 years had positive serology. 57.9% of HCWs who had been vaccinated >10 years before had long-lasting immunity against HBV infection [21].

## 5. Conclusions

From this study, we concluded that at the time of recruitment all laboratory workers should be assessed for the presence of HBsAg and anti-HBsAb titers. All laboratory workers should receive complete three doses of the hepatitis B vaccine and assessed for the anti-HBsAb levels. Those who do not achieve the protective anti-HBsAb titers should be given additional doses. All laboratory workers should then be monitored for their immune status after every five years and booster doses should be administered to those who have become susceptible, since the persistence of protective anti-HBsAb levels is not always related to the number of years elapsed since vaccination.

## Figures and Tables

**Table 1:** Status of HBV vaccination of participants (*n* = 72).

Vaccination status	Number	Percentage
Complete vaccination	34	47.3%
Incomplete vaccination	15	20.8%
Not vaccinated	23	31.9%

**Table 2:** Anti-HBs antibodies status of participants according to vaccination status (*n* = 72).

Vaccination status	Anti-HBsAb titers (mIU/mL)			*P* value (chi-square test)
	<10	>10	% of participants with protective Anti-HBsAb levels	
Complete vaccination	9	25	73.5%	0.032
Incomplete vaccination	7	8	53.3%	
Not vaccinated	14*	9	39.1%	

Total	30	42	58.3%	

*One out of these had acute infection at the time of the study, with negative anti-HBsAb.

**Table 3:** Anti-HBs status of completely vaccinated participants in relation to time since vaccination (*n* = 34).

Vaccination time (years elapsed)	Anti-HBsAb titers ( mIU/mL)			Percentage of subjects with Anti-HBsAb > 100 mIU/mL	*P* value (chi square test)
	<10	10–100	>100		
<5 years (*N* = 2)	0	0	2	100%	
5–10 years (*N* = 17)	7	3	7	41.2%	0.071
>10 years (*N* = 15)	2	8	5	33.4%	

Total	9	11	14	41.2%	

**Table 4:** Status of vaccination in relation to the designation (*n* = 72).

Designation	Complete vaccination (%)	Incomplete vaccination (%)	Not vaccinated (%)	*P* value (chi-square test)
Laboratory Attendants, assistants, and technicians (40)	15 (37.5)	11 (27.5)	14 (35)	0.136
Resident doctors, Ph.D. scholars, and professors (32)	19 (59.4)	4 (12.5)	9 (28.1)	

**Table 5:** Results of hepatitis B serologic tests of all study subjects (*n* = 72).

Serologic test	Result	Number of study subjects (%)	Interpretation
HBsAg Anti-HBsAb Anti-HBcAb Anti-HBeAb	Negative Negative Negative Negative	26 (36.1)	Susceptible

HBsAg Anti-HBsAb Anti-HBcAb Anti-HBeAb	Negative Positive Positive Positive	4 (5.6)	Immune due to recent infection

HBsAg Anti-HBsAb Anti-HBcAb Anti-HBeAb	Negative Positive Positive Negative	3 (4.2)	Immune due to remote infection

HBsAg Anti-HBsAb Anti-HBcAb Anti-HBeAb	Negative Positive Negative Negative	35 (48.6)	Immune due to hepatitis B vaccination

HBsAg Anti-HBsAb Anti-HBcAb Anti-HBeAb	Positive Negative Positive Negative	1 (1.4)	Acutely infected

HBsAg Anti-HBsAb Anti-HBcAb Anti-HBeAb	Negative Negative Positive Negative	3 (4.2)	(1) False positive Anti HBc, therefore susceptible (2) Window period (3) Resolved infection with waning titers of Anti-HBs (4) Occult HBV infection (low levels of HBsAg)
